# Rabbit Enteric Syndrome: The Implications of Rabbit Coronavirus (RbCoV) and Lapine Bocaparvovirus (LBoV) in Rabbitries of Spain

**DOI:** 10.3390/vetsci12111037

**Published:** 2025-10-27

**Authors:** José Luis Arnal, Francisco José Pallarés, Celia Sanz, Carmen Álvarez-Delgado, Gema Chacón, Librado Carrasco

**Affiliations:** 1 Exopol Laboratories, Polígono Río Gállego D/14, San Mateo de Gállego, 50840 Zaragoza, Spain; jlarnal@exopol.com (J.L.A.); csanz@exopol.com (C.S.); gchacon@exopol.com (G.C.); 2 Department of Anatomy and Comparative Pathology and Toxicology, Pathology and Immunology Group (UCO-PIG), UIC Zoonosis y Enfermedades Emergentes ENZOEM, University of Córdoba, International Excellence Agrifood Campus ‘CeiA3’, 14014 Córdoba, Spain; v52aldec@uco.es (C.Á.-D.); librado.carrasco@uco.es (L.C.)

**Keywords:** rabbit, enteric disease, coronavirus, parvovirus, qPCR, histopathology

## Abstract

**Simple Summary:**

Enteric diseases are among the most significant causes of economic losses in the rabbit meat industry, and the role of certain viral agents, such as coronavirus and parvovirus, in their aetiology has not been thoroughly investigated. For this reason, we conducted a study on their detection frequency, associated lesions, and epidemiological patterns to help elucidate the involvement of these viruses in digestive disorders affecting Spanish rabbitries. Coronavirus and parvovirus were consistently detected in farms with recurrent enteric problems and, in most cases, were associated with other bacterial and parasitic agents. Therefore, digestive disorders should be considered a multifactorial condition that requires interventions on multiple fronts, including pathogen control and a thorough review of management and environmental practices.

**Abstract:**

The actual role of certain viruses, such as coronavirus (RbCoV) and parvovirus (LBoV), in the digestive diseases of rabbitries remains poorly understood. For this reason, we conducted three different studies. In the detection frequency study, carried out using samples from both sick and healthy animals from farms with recurrent enteric problems, the presence and Cq values obtained by qPCR for these two viruses supported their implication in digestive disorders. In the lesional study, performed on samples from farms experiencing enteric disease outbreaks, the presence of lesions characteristic of both viruses was confirmed, along with other viral, bacterial, and parasitic agents that may have exacerbated the condition. Finally, in the epidemiological study, using samples from rabbits of different ages from farms with enteric problems, a higher prevalence of these viruses was observed during the growing phase, with a significant association with other bacterial agents such as *Escherichia coli* and *Clostridium spiroforme*. Overall, these results highlight the importance of both viruses in the aetiology of enteric disease and support their inclusion in the diagnostic enteric profile for rabbits.

## 1. Introduction

Digestive disorders are among the most significant causes of economic losses in the rabbit meat industry. These enteric problems primarily affect young animals, including suckling and growing rabbits, leading to a marked increase in morbidity and mortality rates in rabbitries [1,2]. The infectious aetiology is complex and involves multiple bacteria, viruses, and parasites, including enteropathogenic *Escherichia coli* (EPEC), *Clostridium spiroforme*, *Clostridium piliforme*, *Clostridium perfringens*, *Salmonella* spp., *Enterococcus hirae*, *Bacteroides fragilis*, *Eimeria* spp., and lapine rotavirus [1,2]. The development of digestive diseases is influenced by both non-infectious factors, such as housing conditions and nutrition, and infectious agents. Among the latter, viruses such as rotavirus and bacteria including *E. coli*, *C. spiroforme*, and *C. piliforme* are most commonly implicated in these processes [2].

Multifactorial enteropathy, commonly referred to as “enteric syndrome,” significantly affects rabbit intestinal tissues, leading to severe diarrhoea and malabsorption [3]. Diagnosing this condition is often challenging due to the variability of clinical signs and pathological lesions, the concurrent or sequential involvement of multiple pathogenic agents, and the influence of predisposing factors, which frequently hinder rapid and accurate diagnosis.

To date, only lapine rotavirus (rotavirus group A) is widely recognised as a primary viral cause of enteric disease. It is characterised by high morbidity and prominent clinical signs, mainly affecting 4–8-week-old fattening rabbits, although early infection in suckling rabbits (8–21 days old) or late reinfections in 10–12-week-old animals are also possible [3]. Nevertheless, it is generally accepted that other enteric viruses may act as potential pathogens either independently or by facilitating the action of other microbes.

Rabbit coronavirus (RbCoV) was first reported in the 1980s; however, its role has never been fully elucidated [4]. The clinical signs and lesions associated with RbCoV enteric infection are similar to those caused by coronaviruses in other species [3]. Rabbit coronavirus HKU14, a novel *Betacoronavirus* belonging to subgroup A, was isolated from domestic rabbits, and phylogenetic analysis revealed that it is closely related to, yet distinct from, the species *Betacoronavirus 1*, which includes viruses such as bovine coronavirus, equine coronavirus, and porcine haemagglutinating encephalomyelitis virus [5].

Rabbit parvovirus was first described in 1977 [6], and experimental infections in young rabbits demonstrated that it can induce mild clinical signs and mild to moderate catarrhal enteritis [7]. More recently, a novel lapine bocaparvovirus (LBoV) was identified in Italian rabbits, although its role in disease remains uncertain [8]. Despite being considered to have low pathogenic potential, its widespread distribution among rabbitries suggests that it may exert effects in combination with other viral or bacterial agents [3].

Picornavirus has also been detected in the faeces of rabbits from Hungarian farms and has been classified within the genus *Kobuvirus*, designated as rabbit kobuvirus (RKoV) [9]. Infection with this virus is known to cause enteric illness in other mammalian species; however, its role in lagomorphs remains to be clarified. Other viruses, including various caliciviruses [10], adenovirus, reovirus, and enterovirus, have also been reported [3] but are considered to have a minor impact on rabbit enteric diseases.

In this context, the present study was designed to provide a comprehensive assessment of the role of enteric viruses in rabbit enteropathy through three complementary approaches. First, we investigated the relationship between viral presence and the clinical health status of affected animals in order to determine whether infection is consistently associated with overt disease. Second, we explored the association between viral detection and the presence of histopathological lesions compatible with enteric involvement, thereby contributing to a better understanding of the pathological correlations of infection. Finally, we conducted an epidemiological survey in a large population of rabbits affected by enteropathy, with the objective of determining the frequency of these viruses and their interactions with other pathogens that are already well established as part of the rabbit enteric complex. Altogether, these objectives aim to provide new insights into the epidemiology and pathogenesis of enteric viruses in rabbits and to enhance our understanding of their relevance within the multifactorial aetiology of rabbit enteropathy.

## 2. Materials and Methods

### 2.1. Study A: Frequency of Detection of RbCoV, LBoV and RKoB

#### 2.1.1. Animals and Samples

A total of 90 samples, 60 from sick animals and 30 from healthy animals, were collected from rabbitries with recurrent enteric disease problems located in 8 out of the 17 regions of Spain, including the three regions with the highest number of farms: Castilla y León, Cataluña and Galicia [11].

In Spain, commercial rabbit production is generally organised under a continuous band system rather than an “all-in all-out” approach, which is rarely feasible. Breeding units are structured around a stable nucleus of reproductive females that are routinely inseminated artificially. This organisation does give birth and nurse their litters on-site, and kits are weaned at approximately 35 days of age. After weaning, the growing rabbits remain on the same farm throughout the fattening period until they reach market weight and are sent directly to the slaughterhouse. In terms of preventive health management, reproductive females are commonly vaccinated against myxomatosis and rabbit haemorrhagic disease virus (RHDV).

The sampling period extended from 2014 to 2018 and included adult animals (*n* = 4), suckling rabbits (*n* = 15), growing rabbits (*n* = 62), and individuals with missing age data (*n* = 9). Small intestine samples (jejunum and/or ileum) were transported in a thermal container with ice packs to ensure that the temperature remained below 8 °C and were submitted to Exopol Laboratories for real-time PCR (qPCR) analysis to investigate the role of RbCoV, LBoV and RKoV in enteric disorders.

#### 2.1.2. qPCR Detection

First, nucleic acids were extracted and purified using the MagMAX CORE Nucleic Acid Purification Kit (Applied Biosystems, Austin, TX, USA) following the manufacturer’s instructions with the KingFisher Flex device (Thermo Scientific, Rockford, IL, USA). Tissue specimens were pre-treated using the MagMAX CORE Mechanical Lysis Module (Applied Biosystems, Austin, TX, USA) through two runs of 6000 rpm for 30 s in a MagNA Lyser (Roche Diagnostics, Penzberg, Germany).

Three commercial qPCR assays (EXOone series, Exopol, Zaragoza, Spain) were utilised for the detection of RbCoV (ref. RCoV), LBoV (ref. LBoV) and RKoB (ref. RKoB). The assays were performed without biological replicates and were based on hydrolysis probes labelled with FAM fluorophore, targeting RNA-dependent RNA polymerase (*RdRp*) gene in RbCoV and RKoB, and VP1 in LBoV. Simultaneously, the detection of a housekeeping gene within the host cells, using the HEX fluorophore, served as an endogenous control. Thermal profile included an initial enzyme activation step of 5 min at 95 °C, followed by 42 amplification cycles consisting of 15 sec at 95 °C and 60 sec at 60 °C. For RbCoV and LboV detection, since their genomes are RNA-based, a reverse transcription step of 15 min at 45 °C was performed using a one-step protocol.

The qPCR reactions were performed in a QuantStudio 5 Real-Time PCR System (Applied Biosystems, Austin, TX, USA) under the conditions specified by the manufacturer. The results were analysed using QuantStudio software v1.5.2. All results with a cycle threshold of ≤38 were considered positive. Statistical relationships between the presence of each virus and health status were studied through Chi square test. Statistically significant association was assumed in the case of *p*-value < 0.05.

Four samples which had resulted RbCoV positive were selected for *RdRp* gene sequencing to confirm the specificity of the qPCR test. The product of amplification of 440 base pairs was generated with primers and protocol adapted from previous publication [5]. Additionally, the genetic variability of RbCoV strains was studied through *S1* gene from 5 RbCoV positive samples following an adapted protocol and primers described for Bovine Coronavirus [12]. Similarity of the sequences were studied through multiple sequence alignment (MAFFT; version 7) and matrix generated with Sequence Demarcation Tool software (version 1.2).

### 2.2. Study B: Lesional Study

#### 2.2.1. Animals and Samples

Six rabbitries located in the regions of Valencia and Castilla-La Mancha (eastern and central Spain) were included in this study. Their management and rearing conditions were considered similar to those described in Study A. All of them had experienced outbreaks of enteric disease characterised by diarrhoea, dehydration, reduced growth and high mortality. Sampling was carried out entirely in 2021. All affected animals selected for this study belonged to the growing phase, with ages ranging from 42 to 65 days. For each farm, duplicate samples of jejunum and/or ileum were collected from five animals (28 animals in total, since only three animals were sampled in one of the farms). One sample was fixed in 10% neutral-buffered formalin for histopathology, while the other was refrigerated for the comparative qPCR study. The samples were submitted to the Pathology Laboratory of the Faculty of Veterinary Medicine, University of Córdoba, and to Exopol Laboratories, respectively.

#### 2.2.2. Histopathological Study

Four-micron tissue sections from jejunum and/or ileum were stained with haematoxylin and eosin and blindly examined by two pathologists for histopathological evaluation. Four types of lesions were categorised: (a) atrophy and fusion of intestinal villi, (b) vacuolisation of enterocytes, (c) cytomegaly and (d) depletion of Peyer’s patches. The assessment focused on two different processes particularly associated with viral infections: non-regenerative atrophic catarrhal enteritis (NRACE), mostly linked to parvovirus infection due to the involvement of crypt stem cells (a + c), and regenerative atrophic catarrhal enteritis (RACE), which is typically caused by coronavirus and rotavirus, as only the villus epithelial cells show a cytopathic effect (a + b). The presence of other lesion types was also recorded.

#### 2.2.3. qPCR Detection

After nucleic acid extraction, performed as previously described, commercial qPCR assays (EXOone series, Exopol, Zaragoza, Spain) were used for the detection of RbCoV and LBoV, as in Study A, and for the detection of other enteric pathogens, including Lapine rotavirus (ref. RTVA), *Eimeria* spp. (ref. EIME), *Salmonella enterica* (ref. SENT), *B. fragilis* (ref. BFTX), *C. spiroforme* (ref. CSPA), *C. perfringens* (ref. CPTA), and *eae*+ *E. coli* (ref. EEAE). The thermal profile of the qPCR reactions is described in Section 2.2.1, with a distinction between pathogens with RNA genomes, such as RTVA, and those with DNA genomes. Cq values under 38 were considered positive. Statistical relationships between the presence of each virus and health status were studied through Chi square test. Statistically significant association was assumed in the case of *p* < 0.05.

### 2.3. Study C: Epidemiological Situation of RbCoV and LBoV in Spain

The epidemiological situation of RbCoV and LBoV in Spain was investigated through the analysis of 1359 samples of jejunum, ileum and cecum from rabbitries suffering from enteric disease from 15 out of 17 regions of Spain (representing the 96.9% of the total country surface), from years 2020 to 2024. Samples were taken, refrigerated and submitted to Exopol Laboratories for analysis. The samples belonged to adult (*n* = 107), growing (*n* = 885) and suckling (*n* = 233) animals, but in 133 samples the age was not referred. The following pathogens were tested using the qPCR detection protocols described in Studies A and B: LBoV, RbCoV, Lapine rotavirus, *S. enterica*, *eae*+ *E. coli*, *E. hirae*, *Eimeria* spp., *C. spiroforme*, *C. perfringens*, and *B. fragilis*. The normality of Cq values obtained for RbCoV and LBoV was tested using Saphiro-Wilk test. The statistical associations between the presence of both viruses, and between any of them with other agents such as enterotoxigenic *B. fragilis*, *eae*+ *E. coli*, and *C. spiroforme*, were assessed by the Chi-square test. Statistically significant association was assumed in the case of *p* < 0.05.

## 3. Results

### 3.1. Study A: Frequency of Detection of RbCoV, LBoV and RKoB

**Figure 1:**
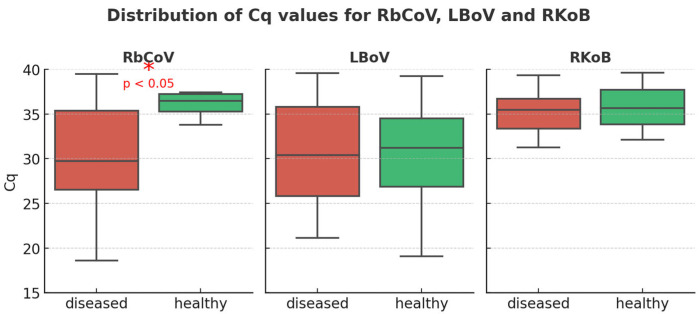
RbCoV, LBoV and RKoB Cq values from diseased and healthy animals from validation collection.

RbCoV was detected in 26/90 (28,88%) samples, 22/60 (36.66%) in sick and 4/30 (13.33%) in healthy rabbits; LBoV was detected in 66/90 samples (73.33%), 46/60 (76.66%) in sick and 20/30 (66.66%) in healthy rabbits; and RKoB was detected in 33/90 (36.66%) samples, 23/60 (38.33%) in sick and 10/30 (33.33%) in healthy rabbits. The Cq values ranged from 18 to 38 for RbCoV and LBoV, whereas they varied from 32 to 38 for RKoB (Figure 1). Statistical association between presence of the virus and disease status was determined only for RbCoV (*p* < 0.05).

The cutoff Cq value was initially set at 38, following the manufacturer’s instructions. However, decreasing Cq thresholds (38, 35, 32, and 30), which corresponded to increasing genomic equivalents of virus per gram of tissue (3 × 10^3^, 3 × 10^4^, 3 × 10^5^, and 1 × 10^6^), were also tested to assess statistical significance. RbCoV invariably obtained highly significant results (*p* < 0.01), while the statistics for LBoV and RKoB were not significant.

The *RdRp* gene sequences obtained (GenBank accession numbers: PV818404 to PV818407) confirmed the specific detection of the RbCoV qPCR test. Additionally, five sequences of the *S1* gene were obtained (GenBank accession numbers: PV818412 to PV818416), showing nucleotide identity values ranging from 70% to 85%.

### 3.2. Study B: Lesional Study

**Figure 2:**
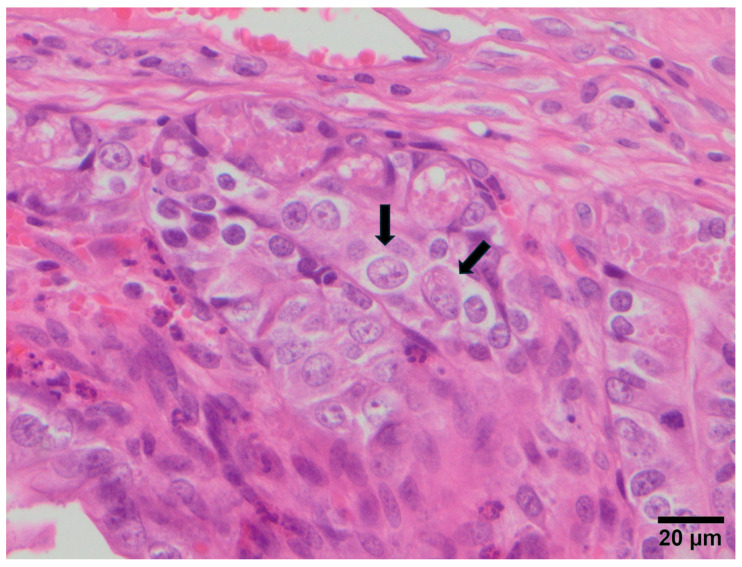
Cytomegaly (arrows) in the crypts of one of the rabbits. NRACE lesion compatible with LBoV infection.

**Figure 3:**
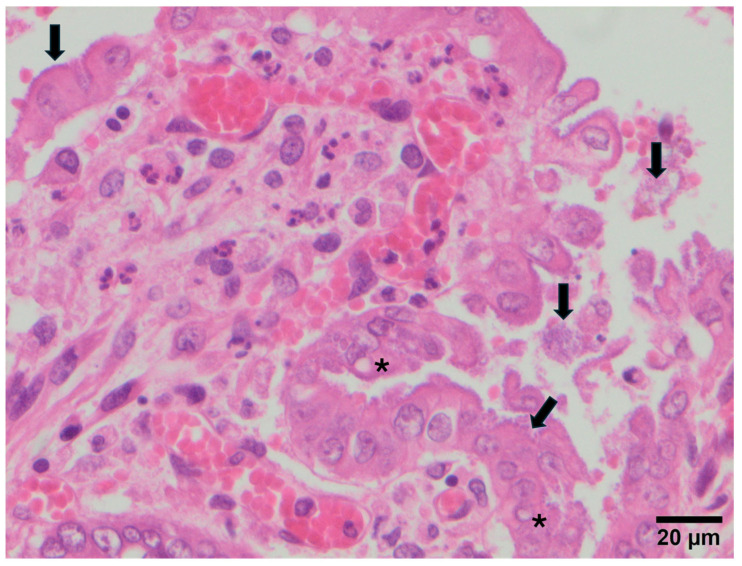
Vacuolisation (asterisks) and detachment of enterocytes and presence of coccobacillary bacteria adhered to them (arrows). RACE lesion compatible with rotavirus/coronavirus infection.

The lesions observed in each animal are summarised in Table 1. Atrophy and fusion of intestinal villi (a) was the most frequent lesion, detected in 20/28 rabbits (71.4%), followed by cytomegaly (c) in 7/28 animals (25.0%) (Figure 2), vacuolisation of enterocytes (b) in 4/28 rabbits (14.3%), and depletion of Peyer’s patches (d) in 2/28 animals (7.1%). In 8/28 samples (28.6%), coccobacillary bacteria adhered to the enterocytes were observed, while coccidia were detected in 1/28 animals (3.6%) (Figure 3).

The combination of atrophy and fusion of intestinal villi with cytomegaly (a + c), indicative of NRACE and compatible with parvovirus (LBoV) infection, was recorded in 7/28 rabbits (25.0%). Likewise, the combination of atrophy and fusion of intestinal villi with vacuolisation of enterocytes (a + b), indicative of RACE and compatible with rotavirus or coronavirus infection, was observed in 4/28 rabbits (14.3%).

Regarding pathogen detection, all pathogens tested were found only in rabbitry 1. LBoV was detected in all rabbitries and in all animals except 1/28 rabbits in rabbitry 3 (R3-1). RbCoV was present in 4/6 rabbitries (1, 2, 3 and 4), although not in all animals. Lapine rotavirus was absent in rabbitries 2 and 4. *C. perfringens* was not detected in rabbitries 3, 4, 5 and 6, and *Eimeria* spp. and *E. coli* (*eae*+) were not found in rabbitries 5 and 6, respectively. Notably, only rabbits R3-5 and R5-5 showed RACE but were negative for lapine rotavirus and RbCoV.

### 3.3. Study C: Epidemiological Situation de RbCoV and LBoV in Spain

**Figure 4:**
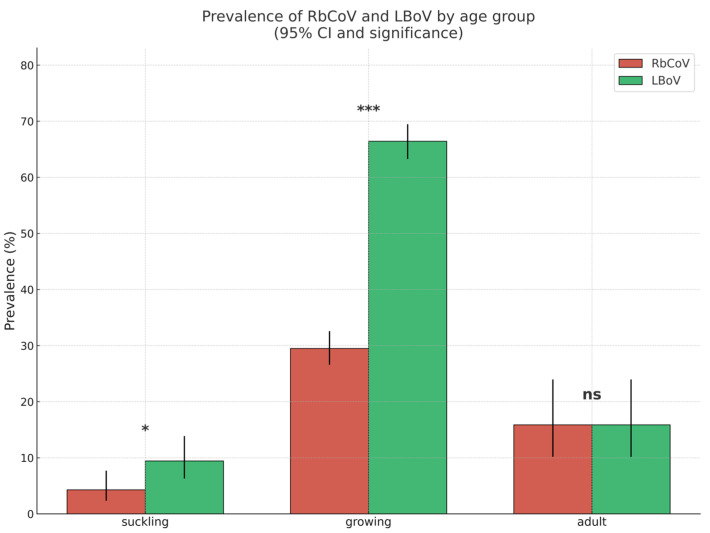
Rate (%) of detection of RbCov and LBoV within different age groups. * *p* < 0.05; *** *p* < 0.005; ns, not significant.

**Figure 5:**
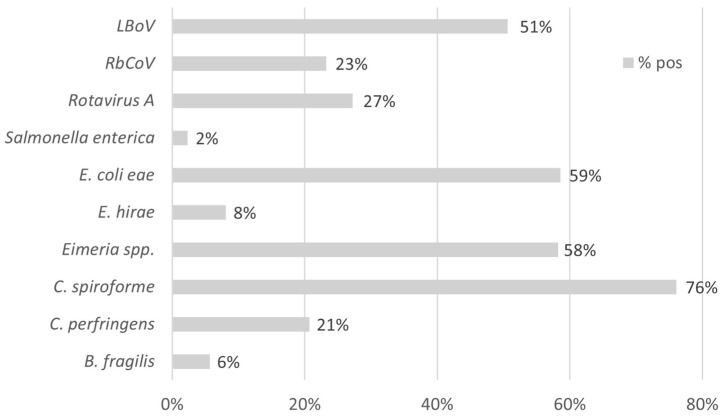
Rate (%) of positive cases of each agent studied within enteric syndrome (*n* = 1359).

The detection rates of RbCoV and LBoV in Spanish rabbitries were 316/1359 (23.25%) and 688/1359 (50.62%), respectively. Growing animals showed notably higher detection rates for both RbCoV and LBoV compared with adults or suckling rabbits, as illustrated in Figure 4. The Cq values for both viruses ranged from 17 to 38, with a median of 29. Neither dataset met the assumption of normality, as indicated by the Shapiro–Wilk test (*p* > 0.05), confirming a non-normal distribution of Cq values. The prevalences of the remaining enteric pathogens tested in the syndrome panel are presented in Figure 5.

A significant association (*p* < 0.05) was found between the presence of RbCoV and LBoV. In addition, RbCoV showed a statistically significant association with *eae*+ *E. coli* (*p* < 0.05), while LBoV exhibited significant relationships with *B. fragilis* (*p* < 0.001), *eae*+ *E. coli* (*p* < 0.05) and *C. spiroforme* (*p* < 0.05).

## 4. Discussion

Enteric problems are a major cause of economic losses in rabbitries due to growth retardation and increased clinical signs and mortality, and viruses are among the main pathological agents involved in these conditions [1,2]. The role of viruses such as lapine rotavirus (Rotavirus group A) as a primary cause of enteric disease in suckling and fattening rabbits is well established [3], but the real prevalence and effect of other potential viral agents such as RbCoV, LBoV or RKoV in digestive problems have not been fully described. Accordingly, this work aimed to determine the presence of RbCoV, LBoV and RKoV in animals from Spanish commercial rabbitries and to assess their relevance in digestive disorders using a combination of molecular detection methods and histopathological studies.

The frequency of detection of these three viral agents was investigated in samples from rabbitries with recurrent enteric disease problems from different regions of Spain, including the regions with the highest number of farms and animals (Study A). The highest detection rate was observed for LBoV (66/90, 73.33%), followed by RKoV (33/90, 36.66%) and RbCoV (26/90, 28.88%), and rates were consistently higher in sick than in healthy rabbits (76.66% vs. 66.66% for LBoV, 38.33% vs. 33.33% for RKoV, and 36.66% vs. 13.33% for RbCoV). A similar detection rate of LBoV (75%) was reported in laboratory rabbits in the United States by Metcalf et al. [13] using serology, whereas a lower prevalence (46.7%) was observed in commercial farms in Japan by Matsunaga et al. [6], also using serology. For RbCoV, a serological study conducted in the United States and Canada in six rabbitries reported detection rates ranging from 3% to 40%, with most seropositive rabbits older than four months [14]. Information on the prevalence of RKoV is scarce, but PCR-based studies in faecal and blood samples reported a prevalence of 28.6% in faeces in commercial farms in Hungary [9] and values of 23.68% in faeces and 18.4% in blood in experimental rabbits in China [15], which are slightly lower than the detection rate of RKoV observed by qPCR in our study. In Study A, Cq values of positive samples for LBoV and RbCoV ranged from 18 to 38, while RKoV showed Cq values from 32 to 38, indicating a higher viral load for the first two agents.

Based on previous serological and qPCR studies from other countries, the greatest enteric pathogenic impact in Spanish rabbitries was considered more likely for LBoV and RbCoV, although a significant association between viral presence and disease status was only found for RbCoV (*p* < 0.05). Because the difference in detection rate between healthy and sick animals observed for RKoV was very small and the Cq values obtained were high, the pathogenic impact of this virus in Spanish rabbitries was considered limited, and RKoV was therefore not included in the lesional and epidemiological studies.

As a significant association between viral presence and disease status was only found for RbCoV, *RdRp* gene sequences were obtained exclusively for this virus. The identity values obtained from the *S1* gene sequences (70–85%) indicate considerable genetic diversity and suggest a long period of evolution since its introduction. Possible recombination events during evolution through cross-species transmission have been suggested by Lau et al. [5] for this virus.

In the lesional study (Study B), lesions compatible with NRACE, indicative of LBoV infection, were detected in five out of six farms, and the virus was detected by qPCR in 27/28 animals (96.42%). These findings corroborate the high detection rate recorded for this pathogen in Study A and its relevance in enteric disease in Spanish rabbitries. In contrast, lesions compatible with RACE, indicative of RbCoV infection, were found in four out of six farms and in 12/28 animals (42.85%). However, in three rabbitries (farms 3, 5 and 6), the affected rabbits were negative for RbCoV by qPCR, and only in one rabbit from farm 6 was lapine rotavirus, which causes similar lesions, detected, indicating that other pathogens could contribute to the development of RACE in these rabbits.

All rabbits that tested positive for RbCoV by qPCR, except for one animal from farm 1, were also positive for LBoV. In a survey conducted by Cerioli and Lavazza [3] on rabbits with enteritis by electron microscopy between 2002 and 2005, the association parvovirus + coronavirus was found in 17.39% of samples, parvovirus + rotavirus in 4.34%, and coronavirus + rotavirus in 39.13%. In our study, these associations were identified in 12/28 (42.85%), 9/28 (32.14%) and 3/28 (10.71%) of samples, respectively (Table 1). Differences may be related to variation in the sensitivity and specificity of the diagnostic techniques employed. The presence of the LBoV–RbCoV combination appears relevant in the enteric disorders of Spanish rabbitries, consistent with observations from other countries such as Italy [3].

Enteropathogenic *E. coli* causes significant diarrhoea-associated morbidity in rabbits and has zoonotic potential [16]. In Study B, *eae+ E. coli* was the most prevalent bacterial agent detected, present in five out of six farms and in 23/28 animals (82.14%). *C. spiroforme* is considered the most common clostridial pathogen in rabbit enterotoxaemia, and *C. perfringens* may also be involved [17]. In our study, *C. spiroforme* was detected in all farms and in 18/28 rabbits (64.28%), while *C. perfringens* was limited to two farms and two animals (7.14%). Coccidiosis caused by *Eimeria* spp. infection can be a serious problem and a significant cause of economic losses in rabbit farms [18], and in our study this protozoan was detected in five out of six farms and in 11/28 animals (39.28%). These bacteria and protozoa were consistently present in animals positive for LBoV, and coinfections were frequent.

The purpose of Study C was to determine the epidemiological situation of Spanish rabbitries with respect to RbCoV and LBoV through a molecular study of samples collected from animals with enteric problems from farms across almost the entire national territory, differentiating between suckling, growing and adult animals. The overall prevalence of both viruses in the country showed a similar trend to the detection frequency observed in the selected farms in Study A, with a higher prevalence of LBoV (688/1359, 50.62%) than RbCoV (316/1359, 23.25%). These values, together with the recorded Cq range (17–38, median 29), support the importance of both viruses in the aetiology of enteric disorders in Spanish rabbitries and are in line with previous observations based on electron microscopy [3] and serological techniques [14].

When prevalence was analysed by production phase, both viruses showed higher prevalence in growing rabbits, followed by adults and suckling animals. The difference in favour of LBoV was more notable in growing rabbits, less evident in suckling animals and similar in adults. These data are consistent with observations indicating that viral enteric problems on farms are more significant during the lactation and growing periods and less significant in adult animals [2,19,20].

The association study among the different pathogens confirmed the significant association between RbCoV and LBoV, also observed in the lesional study (Study B). The associations between these viruses and other bacterial agents such as *E. coli*, *B. fragilis* and *C. spiroforme*, also detected in Study B, indicate that coinfection with viral and non-viral pathogens is common in commercial rabbitries and can contribute to more severe disease than viral infection alone [3,19,21]. Consequently, enteric disorders in rabbitries should be addressed as a complex process in which, in addition to pathogens, environmental conditions and management practices should be considered for effective control.

This study has several limitations that should be acknowledged. Although whole genome sequencing (WGS) could have provided valuable information to further explore potential recombination events among circulating strains, this approach was not feasible due to limited resources. Moreover, the generation of complete viral genomes would probably have required prior viral isolation of field strains, a resource that was not available. WGS-based genetic analyses could also have supported evolutionary studies of circulating strains. Such an approach, particularly when applied to a large sample set with broad temporal and geographical coverage, could yield valuable insights into the introduction and evolution of different strains within our country, thereby enabling a more in-depth discussion. Similarly, in situ labelling techniques could have provided additional evidence to better confirm the role of the studied viruses within the enteric disease complex; however, this approach could not be implemented due to the lack of specific antibodies.

Moreover, the high number of pathogens associated with enteric disease in rabbits makes it extremely difficult to describe the virulence of a single virus per se, isolating its effects from those of other agents. Experimental infections using specific-pathogen-free animals could yield further insights, but would hardly reflect the real epidemiological situation, in which farmed rabbits are continuously exposed to coinfections. In field conditions, with samples obtained from fattening animals, it remains challenging to precisely define the primary or secondary role of these viruses, as well as to characterise the temporal progression of successive infections. For these reasons, our work was primarily descriptive, emphasising the detection and presence of RbCoV, LBoV, and RKoB in Spanish rabbitries and reporting the most frequent coinfections across production stages.

While advanced techniques such as electron microscopy, viral isolation, or WGS would certainly have strengthened the conclusions, the main aim of the present study was to provide molecular evidence of these viruses and to describe compatible histological lesions. Future studies integrating those advanced techniques will be essential to clarify the epidemiology frame and pathogenic role of these agents within the multifactorial enteric disease complex of rabbits.

## 5. Conclusions

This research demonstrates the frequent infection of RbCoV and LBoV in commercial rabbitries in Spain. The association of RbCoV with enteric disease and the description of compatible lesions indicate a potential primary role of this virus in the onset of illness. LBoV, in turn, shows high prevalence rates, compatible histopathological findings and statistical associations with other pathogens such as *E. coli*, *C. spiroforme* and *B. fragilis*, which are accredited agents within the enteric syndrome. These observations support the inclusion of both viruses in the diagnostic digestive profile of rabbits. Future research should focus on clarifying the pathogenic mechanisms of RbCoV and LBoV and on evaluating the efficacy of control strategies such as regular or autogenous vaccination and specific biosecurity measures.

## Figures and Tables

**Table 1:** Microscopic lesions and results of qPCR for all pathogens tested in each animal.

	HP	qPCR						
Rabbitry and Sample	Lesion	LBoV	RbCoV	Lapine Rotavirus	*Eimeria* spp.	*C. spiroforme*	*C. perfringens*	*E. coli (eae)*
R1-1	a + c (NRACE)	35.27	21.02	N/A	N/A	30.33	N/A	21.41
R1-2	a + c (NRACE)	25.77	27.68	34.89	33.99	36.54	N/A	22.63
R1-3	d	24.09	39.26	32.95	36.09	N/A	N/A	21.15
R1-4	d	34.18	34.15	N/A	N/A	N/A	36.99	21.73
R1-5	a	22.90	24.44	N/A	N/A	34.07	N/A	22.53
R2-1	a + c (NRACE)	30.78	30.99	N/A	35.01	N/A	N/A	20.38
R2-2	W/A	20.92	N/A	N/A	31.09	29.51	36.27	36.38
R2-3	a	20.28	N/A	N/A	33.15	29.83	N/A	N/A
R2-4	a	35.11	N/A	N/A	28.58	31.86	N/A	22.89
R2-5	a	29.66	N/A	N/A	31.92	N/A	N/A	N/A
R3-1	a + c (NRACE)	N/A	38.11	38,63	N/A	33.49	N/A	20.92
R3-2	W/A	36.99	32.37	35.37	N/A	N/A	39.93	21.90
R3-3	a	31.25	35.09	37.71	31.04	N/A	N/A	22.75
R3-4	a + c (NRACE)	36.76	38.50	N/A	35.91	N/A	38.67	20.17
R3-5	a + b (RACE)	29.28	38.81	39,72	N/A	33.09	N/A	23.73
R4-1	a + c (NRACE)	33.97	36.38	N/A	37.61	29.40	N/A	23.30
R4-2	W/A	37.91	35.50	N/A	N/A	35.02	N/A	22.85
R4-3	a	33.22	25.96	N/A	N/A	N/A	N/A	23.04
R4-4	W/A	29.40	35.13	N/A	N/A	32.13	N/A	23.08
R4-5	a + b (RACE)	28.24	32.07	N/A	N/A	37.70	N/A	26.90
R5-1	a	25.30	N/A	36.44	N/A	29.74	N/A	21.58
R5-2	W/A	29.64	N/A	38.19	N/A	34.20	N/A	20.25
R5-3	a + c (NRACE)	27.85	N/A	39.68	N/A	N/A	N/A	21.29
R5-4	a	36.74	N/A	36.66	N/A	N/A	N/A	20.12
R5-5	a + b (RACE)	35.18	N/A	N/A	N/A	29.17	N/A	19.12
R6-1	a	32.55	N/A	28.77	N/A	29.10	38.26	N/A
R6-2	a + b (RACE)	23.89	N/A	29.25	N/A	27.45	N/A	N/A
R6-3	W/A	34.02	N/A	35.86	31.60	23.71	N/A	N/A

Rx-x: Rabbitry number and number of samples in each one; HP: histopathology; LBoV: Lapine Bocaparvovirus; RbCoV: Rabbit Coronavirus; a: atrophy and fusion of intestinal villi; b: presence of vacuoles in enterocytes; c: cytomegaly; d: depletion of Peyer’s patches; W/A: without microscopic alterations; N/A: not amplify. Table cells in green: positive result by qPCR; Table cells in red: negative result by qPCR.

## Data Availability

The original contributions presented in this study are included in the article. Further inquiries can be directed to the corresponding author.

## Abbreviations

The following abbreviations are used in this manuscript:

RbCoV	Rabbit Coronavirus
LBoV	Lapine Bocaparvovirus
RKoB	Rabbit Kobuvirus
qPCR	Real-time PCR
*RdRp*	RNA-dependent RNA polymerase
NRACE	Non-Regenerative Atrophic Catarrhal Enteritis
RACE	Regenerative Atrophic Catarrhal Enteritis
WGS	Whole Genome Sequencing
